# Hypertonic Saline Attenuates the Pro-metastatic Effects of LPS by Reducing Tumor Cell Migration, Proliferation and MMP-9 Expression

**DOI:** 10.4021/wjon420w

**Published:** 2011-12-19

**Authors:** Mark Corrigan, Conor Shields, Donald O'Leary, John Fraher, Desmond Winter, Jianghuai Wang, Paul Redmond

**Affiliations:** a Departments of Surgery and Anatomy, University College Cork, Ireland; b Department of Surgery, St. Vincent’s University Hospital, Dublin, Ireland

**Keywords:** Metastases, Hypertonicity, Lipopolysaccharide

## Abstract

**Background:**

Lipopolysaccharide (LPS) promotes tumor metastases. The aim of this study was to determine the ability of a hypertonic environment to attenuate the pro metastatic properties of LPS both in vitro, and in vivo.

**Methods:**

LPS stimulated, and unstimulated, 4T1 tumor cells were cultured in either an isotonic or hypertonic environment. The effect on invasion, migration, pro-matellomatrixproteinase 9 (proMMP-9) expression, proliferation, and microscopic cell structure was assessed. Lung metastases were induced in C57 mice with systemic hypertonicity in unstimulated and stimulated mice. The metastatic burden was assessed by estimation of lung/body weight ratio, pleural nodules and clonogenic assay.

**Results:**

In vitro, a hypertonic environment reduced proMMP-9 expression (0.012 versus 1.16, P < 0.001) invasion (0.06 versus 0.119, P = 0.005), tumor cell proliferation (0.035 versus 0.041, P = 0.001), while inducing structural changes to tumor cells reducing overall cell volume. In vivo, the induction of transient systemic hypertonicity reduced metastatic burden as demonstrated by reduced lung nodules (4 versus 8, P = 0.004) and colonies on clonogenic assay (12 versus 43, P = 0.04).

**Conclusion:**

The in vitro exposure of tumor cells to a hypertonic environment reduces tumor cell migration and proliferation. Transient systemic hypertonicity can reduce the metastatic burden following intra-operative exposure to LPS in vivo.

## Introduction

A combination of proliferation, migration and adhesion are the key properties which confer tumor cells motility [1]. Previous studies have demonstrated an impaired adhesive ability in tumor cells exposed to a hypertonic environment [2], suggesting a potential tumor impeding effect. Little is known of the effect of hypertonicity on the structure of solid tumor cells. Any mechanical changes may be of importance in explaining the shedding of adhesion molecules seen in hypertonic environments [3] and as a consequence its effect on cell proliferation [3, 4]. Furthermore, the degradation of extra cellular proteins removes physical barriers, through the actions of matrix metalloproteinases (MMPs), allowing the passage of malignant cells across the basement membrane. MMP-9 is an important subtype of at least 23 secreted and membrane-bound zincendopeptidases. In particular MMP-9 is important in the progression of several different tumor types [5-8] and is thought to be one of the key factors related to increased tumor growth post surgical insult [9].

Although the mainstay of oncologic treatment, surgical resection has the potential to encourage both tumor growth and metastases [9-11]. Several factors may be involved in this phenomenon. It is known that the process of tumor excision profoundly effects the properties of existing tumor cells, reducing apoptosis and increasing proliferation [12]. This is mediated through the inflammatory response which can encourage tumor growth [13, 14]. It has been postulated that this is mediated by blood borne factors such as lipopolysaccharide (LPS). LPS increases metastatic burden [12, 15, 16], an affect which appears to be mediated through a combination of angiogenesis [17] and the up-regulation of adhesion factors [18]. Additionally, LPS results in an increase in tumor cell adhesion via an increase in β1-integrin expression [19].

The anti-inflammatory effects of transient systemic hypertonicity, through use of hypertonic saline (HTS), has been demonstrated in several animal models, including attenuating the inflammatory cascade associated with bacterial challenge [20], lung injury [21], ischemia reperfusion [21], and pancreatitis [22]. Furthermore the safety of HTS has been established in human trials as a potential resuscitation fluid following trauma [23, 24].

This study sought to determine if a hypertonic environment can reduce the invasion and migration of tumor cells in vitro and attenuate the metastatic promoting properties of LPS. In addition, it sought to determine if the induction of a transient systemic hypertonicity can reduce the metastatic load following tumor cell dissemination and LPS exposure in an in vivo model.

## Materials and Methods

### Solutions

Standard culture medium (DMEM) along with isotonic saline solutions containing NaCL (osmolality 320 mosmol), were used as controls. In order to replicate the clinical environment, hypertonic saline solutions contained an additional 50 mM NaCL giving an osmolality of approximately 415mosmol. This osmolality is similar to the initial effects of an in vivo infusion of 7.5% NaCL [25].

### Cell culture

The murine breast cancer cell line 4T1 was used in all experiments. This was maintained in Dulbecco modified Eagle medium (DMEM) (BioWhittaker, Europe), which was supplemented with 10% fetal calf serum, penicillin (100 units/mL) and streptomycin (100 µL/mL), amphotericin B (0.25 Lg/mL) and glutamine (2 mmoL/L). The cells were grown at 37 °C in a humidified atmosphere with 5% CO_2_. The medium was renewed 3 times weekly. Only single-cell suspensions of greater than 90% viability as determined by Trypan Blue exclusion were used for injections.

### Determination of tumor cell invasion through an extra cellular matrix layer

Assessment of tumor cell invasion was based on the Boyden chamber principle using the fluorescent CyQuant GR dye (Chemicon, Linco, USA) in accordance with the manufacturer’s instructions. The results were read with a fluorescence plate reader using 480/520 nm filter set (Thermolabsystems, Franklin, USA).

### Determination of pro-Metalloproteinase 9 in cell culture supernatants

Cellular pro-MMP-9 expression was quantified using a quantitative sandwich enzyme immunoassay technique (R&D Systems, Minneapolis, USA) in accordance with the manufacturer’s instructions. The optical density of each well was measured using a microplate reader set to 450nm and wavelength correction set to 540 nm (Thermolabsystems, Franklin, USA). Sample values were then read off the standard curve with measurements given in ng/mL.

### Determination of tumour cell proliferation in a hypertonic environment

Tumour cell proliferation was quantified using a BrdU cell proliferation ELISA (Roche Diagnostics). Briefly, tumour cells (1 x 10^4^ cells) were cultured in 96 well plates for 12 hours in either culture medium, normal saline or a hypertonic environment, with or without LPS at 100 ng/mL (Sigma Corp, Dublin, Ireland) at 37 °C in a humidified 5% carbon dioxide atmosphere. These groups were duplicated in the presence of LPS 100 ng/mL. After 24 hours BrdU was added to the cells and the cells were incubated for 12 hours allowing the pyrimidine analogue BrdU to be incorporated in place of thymidine into the DNA of proliferating cells. After 12 hours the culture medium was removed, the cells fixed and the DNA denatured. Next the anti-BrdU-POD monoclonal antibody was added binding to the BrdU incorporated in newly synthesized, cellular DNA. After washing, a substrate solution was added and the resulting reaction product was quantified by using a scanning multiwall spectrophotometer at 450 nm.

### Assessment of apoptosis

Cells were then plated in either culture medium, normal saline or a hypertonic environment, with or without LPS at 100 ng/mL (Sigma Corp, Dublin, Ireland) at 37 °C in a humidified 5% carbon dioxide atmosphere. Determination of apoptosis of both quiescent and stimulated cells by flow cytometry (BD FACSCalibur, New Jersey, USA) was performed after 1 and 12 hours. Apoptosis was quantified according to the percentage of cells with hypodiploid DNA by the use of the propidium iodide staining technique as previously described [26]. All measurements were performed using the same instrument settings.

### Cell viability

Cell viability was assessed by measuring the ability of cells to metabolize MTT (Sigma-Aldrich, St. Louis, USA); a water-soluble tetrazolium salt, into a water-insoluble formazan product as previously described [27]. The fluorescence was measured in each well using a plate reader (Thermolabsystems, Franklin, USA) at an absorption wavelength of 560 nm.

### The effect of a hypertonic environment on the cellular structure of tumor cells

The effect of increased tonicity on tumor cell structure was assessed using transmission electron microscopy. Briefly, 1 x 10^5^ cells in 3ml of culture medium were inoculated into each well of a 12-well plate suitable for electron microscopic studies (Transwell®, St. Louis, USA) and further cultured for 24 hours. After 24 hours the culture medium was removed and the cell monolayer washed three times with PBS. Cells were then cultured for a further 1 hour in either hypertonic medium, isotonic medium or in normal saline/culture medium. These groups were duplicated in the presence of LPS at a concentration of 100 ng/mL. Following this the cell medium was removed and the cells processed for electron microscopic assessment. Cells were fixed in “primary” fixative for 1 hour (2% glutaraldehyde, 2.5% paraformaldehyde, 0.165 molar phosphate buffered, pH 6.8). Specimens were then immersed in 0.165 molar phosphate buffer for 20 minutes, followed by treatment in “secondary” fixative for one hour (2% Osmium tetroxide in 0.165 molar phosphate buffer). As a control a separate set of specimens were processed under hypertonic conditions. Solutions for those hypertonic cells were adjusted to 420 milliosmoles using Sodium Chloride. All specimens were then dehydrated in a series of ethanols. They were then left overnight in a mixture of 3 parts araldite to one part propylene oxide. Specimens were then immersed in araldite for 6 hours before immersion in final araldite, and incubated in an oven at 50 degrees centigrade for 48 to 72 hours where polymerization occurs. Specimens were semithin sectioned (approximately 0.5 micron thickness) and stained with a solution of 1% toluidine blue in 1% borax for light microscopy. They were then ultra-thin sectioned (about 120 nm. thickness) for transmission electron microscopy. Glass knives, for semithin sectioning, were made with a Leica EM KMR2 knife maker (Leica, Wetzlar, Germany). A Diatome diamond knife was used for ultra-thin sectioning. Semithin and ultra-thin sectioning was performed with a Reichert-Jung Ultracut E ultramicrotome (Leica, Wetzlar, Germany). Ultra-thin sections were stained with heavy metal stains, firstly with 2% uranlyl acetate in methanol; secondly with Lead Citrate. Finally the specimens were examined and photographed using the transmission electron microscope (Jeol JEM-2000FXII, Tokyo, Japan), with a digital camera (Olympus, MegaView III Soft Imaging System, Tokyo, Japan). Analysis of the images was performed in a blinded fashion using previously described stereological methods [28].

### Experimental model

Lung metastases were induced using a model previously described [29]. Animals were sedated, using intraperitoneal Hypnorm® (VetaPharm Ltd, Suffolk, UK). Mice received 5 x 104 4T1 cells in 100 µL PBS via lateral tail vein injection.

In the first experiment, 20 mice received an intravenous bolus of 2 mL/kg normal saline (0.9% sodium chloride; NS) (n = 10) or 2 mL/kg hypertonic saline (7.5% sodium chloride; HTS) (n = 10) via lateral tail vein, 2 minutes after tumor injection. After 14 days, all mice were sedated, weighed and euthanized by cervical dislocation. Lungs were harvested and lung weight and incidence of surface pleural r lesions recorded. Number and size of experimental metastases was determined by an independent observer and the number of clonogenic lung metastases was determined.

In a second experiment to determine the effect of LPS in the presence of systemic hypertonicity, 20 mice received an intraperitoneal (i.p.) injection of 10 µg lipopolysaccharide (serotype 055:B5; Sigma Chemical, St. Louis, MO) 7 days after tumor injection. Two minutes later systemic hypertonicity was induced with a bolus of 2 mL/kg normal saline (0.9% sodium chloride; NS) (n = 10) or 2 mL/kg hypertonic saline (7.5% sodium chloride; HTS) (n = 10). All animals were warmed until recovered from sedation. After 14 days, all mice were sedated, weighed and euthanized by cervical dislocation. Lungs were harvested and lung weight and incidence of surface pleural lesions recorded. The number and size of experimental metastases were determined by an independent observer and the number of clonogenic lung metastases was determined.

### Clonogenic assay

The numbers of metastatic 4T1 cells in lungs were determined by clonogenic assay [30]. In brief, on day 14, the lungs were removed from each mouse, finely minced, digested in 5 mL of enzyme mixture containing 1 x PBS and 1 mg/mL collagenase type IV, and incubated for 2 h at 37 °C on a platform rocker. After incubation, samples were filtered through 70 µm nylon cell-strainers and washed twice with PBS. The resulting cells were suspended, plated with a series of dilutions in 10 cm tissue-culture dishes in RPMI medium 1640 containing 60 µm thioguanine for clonogenic growth. Because 4T1 tumor cells are resistant to 6-thioguanine, metastasized tumor cells formed foci after 14 days, at which time they were fixed with methanol and stained with 0.03% methylene blue for counting.

### Data analysis

All parametric, in vitro data, are presented as mean values with standard error of mean (SEM). Statistical analysis was performed using ANOVA, and a p value < 0.05 was considered significant. Non parametric, in vivo data, are presented as median values, and a p value of > 0.05 was considered significant.

## Results

### Determination of tumor cell invasion through an extra cellular matrix layer

**Figure 1:**
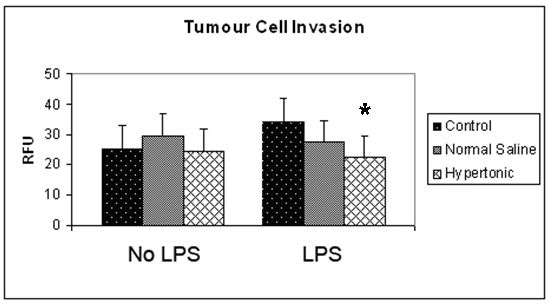
Effect of hypertonicity on tumor cell invasion. Tumor cells were exposed to hypertonic or isotonic conditions in the presence or absence of LPS before quantitative determination of tumor cell invasion at 12 hours. Data expressed as mean and are representative of 8 separate experiments. (^*^P < 0.05 versus isotonic culture medium and LPS)

It was observed that exposure to a hypertonic environment did not significantly affect the invasive capacity of unstimulated 4T1 tumor cells (Fig. 1). However, exposure of 4T1 cells to 100 ng/mL LPS increased the ability of 4T1 cells to invade through an extra cellular matrix layer. This affect was attenuated by a hypertonic environment which reduced invasion post stimulation from 34.5 to 22.1 RFU (P = 0.01).

### Determination of pro-Metalloproteinase 9 in cell culture supernatants

**Figure 2:**
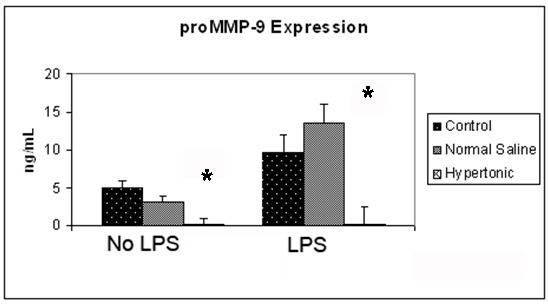
Effect of hypertonicity on tumor cell expression of proMMP-9. Tumor cells were exposed to hypertonic or isotonic conditions before quantitative determination of proMMP-9 expression at 12 hours. Data expressed representative of 8 separate experiments. ^*^P < 0.05 versus isotonic culture medium.

The expression of proMMP-9 in 4T1 cells was assessed at 12 hours, both in the presence and absence of LPS. Exposure to a hypertonic environment significantly reduced the expression of proMMP-9 from 4.95 ng/mL to 0.07 ng/mL (P > 0.001). Exposure of 4T1 cells to the pro-inflammatory mediator LPS, resulted in a significant increase expression of proMMP-9, doubling the original expression. This effect was attenuated by hypertonic exposure, reducing proMMP-9 expression to almost negligible levels from 9.49 ng/mL to 0.1 ng/mL (P ≤ 0.001) (Fig. 2).

### The effect of a hypertonic environment on the morphology of tumor cells

**Figure 3:**
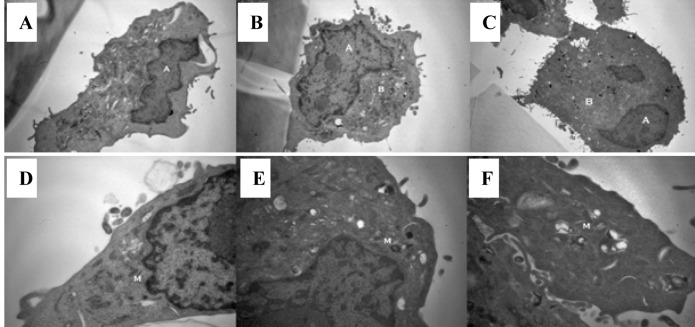
Exposure to a hypertonic environment (B) results in a reduced nuclear to cytoplasmic ratio, when compared to isotonic culture medium (A) or isotonic normal saline (C): magnification x 8000. Furthermore, a hypertonic environment results in a decrease in the electron density of cellular mitochondria (E) compared to those cultured in isotonic culture medium (D) or isotonic normal saline (F): magnification x 20 000.

Tumor cell morphology was visualised through use of transmission electron microscopy. Cells were imaged following 1 hour exposure to a hypertonic environment. Two groups exposed to culture medium and normal saline were used as controls. The cells in a hypertonic environment exhibited a reduction in nuclear/cytoplasmic ratio (P = 0.005) (Fig. 3A-C). Cells exposed to a hypertonic environment possessed well defined plasma membranes and contained intact organelles with no evidence of nuclear condensation or apoptotic bodies. No differences in vacuoles, lysosomes or secretions were noted between the groups. Mitochondria from those cells cultured in a hypertonic environment were less electron dense suggesting an increased physiological strain on the cell (Fig. 3D-F).

### Determination of tumour cell proliferation in a hypertonic environment

Tumour cell proliferation was assessed in both isotonic and hypertonic environments. It was observed that exposure to a hypertonic environment resulted in a significantly reduced proliferation of 4T1 tumour cells (0.035 versus 0.041, P < 0.001). Interestingly exposure of tumour cells to normal saline resulted in a significant increase in tumour cell proliferation (P = 0.04). When the effect of LPS on tumour cell proliferation was assessed at a concentration of 100 ng/mL, no significant change in tumour cell proliferation could be demonstrated.

### Effect of hypertonicity on the apoptotic rate and viability of 4T1 cells

In order to ensure our findings were not due to increased apoptosis, the apoptotic rate was quantified at 1 and 12 hours. Exposure to a hypertonic environment did not significantly change the rate of apoptosis or the cell viability after 1 or 12 hours. However the exposure of 4T1 cells to 100 ng/mL LPS for 1 hour resulted in significantly decreased cell viability (56% decreased viability P = 0.008). This appears to be a transient effect, with some recovery of cell viability evident after 12 hours exposure to LPS (34% decreased viability P = 0.143). The exposure of 4T1 cells to a hypertonic environment in the presence of LPS did not further affect the cell viability.

### The effect of systemic hypertonicity on the metastatic burden following tumor cell dissemination

**Figure 4:**
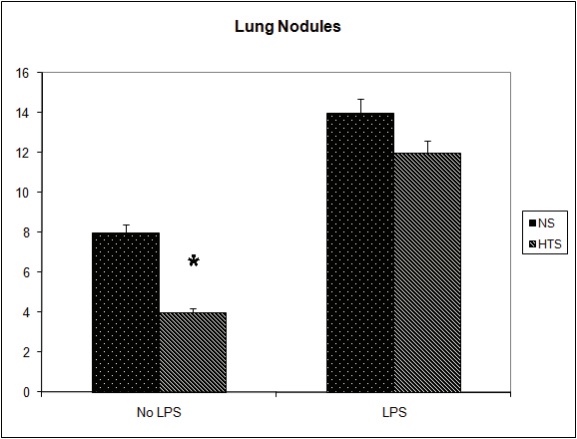
Effect of hypertonicity on lung nodules. Animals were injected with 5 x 10^4^ 4T1 tumor cells. Two minutes later mice received an intravenous bolus of 2 mL/kg normal saline (0.9% sodium chloride; NS) or 2 mL/kg hypertonic saline (7.5% sodium chloride; HTS) via lateral tail vein. Animals were sacrificed after 14 days and lung nodules counted. In a second experiment Effect of hypertonicity on lung/body weight ratio (g/kg) following LPS stimulation. Animals were injected with 5 x 10^4^ 4T1 tumor cells. Seven days later mice received an intraperitoneal (i.p.) injection of 10 µg lipopolysaccharide. Two minutes later the animals received either an intraperitoneal bolus of 2 mL/kg normal saline (0.9% sodium chloride; NS) or 2 mL/kg hypertonic saline (7.5% sodium chloride; HTS) via lateral tail vein. Animals were sacrificed after 14 days and lung nodules counted. Data are expressed as mean and are representative of n = 20 animals. ^*^P < 0.05 versus NS control.

**Figure 5:**
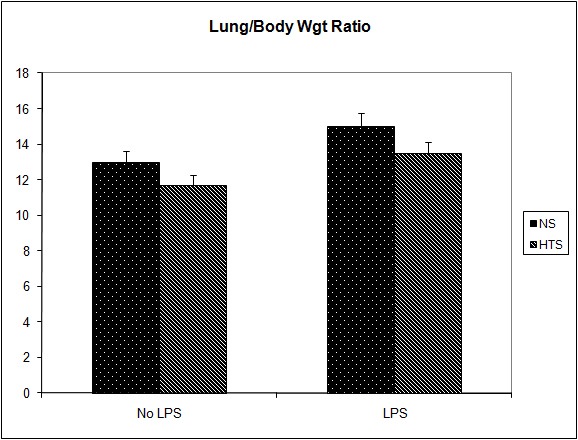
Effect of hypertonicity on lung/body weight ratio (g/kg). Animals were injected with 5 x 10^4^ 4T1 tumor cells. Two minutes later mice received an intravenous bolus of 2 ml/kg normal saline (0.9% sodium chloride; NS) or 2 mL/kg hypertonic saline (7.5% sodium chloride; HTS) via lateral tail vein. Animals were weighed and sacrificed after 14 days and lungs weighed to calculate lung/body weight ratio. In a second experiment to determine the effect of hypertonicity on lung/body weight ratio (g/kg) following LPS stimulation, animals were injected with 5 x 10^4^ 4T1 tumor cells. Seven days later mice received an intraperitoneal (i.p.) injection of 10 µg lipopolysaccharide. Two minutes later the animals received either an intraperitoneal bolus of 2 mL/kg normal saline (0.9% sodium chloride; NS) or 2 mL/kg hypertonic saline (7.5% sodium chloride; HTS) via lateral tail vein. 7 days later animals were weighed and sacrificed and their lungs weighed to calculate lung/body weight ratio. Data are expressed as mean and are representative of n = 20 animals. ^*^P < 0.05 versus NS control.

**Figure 6:**
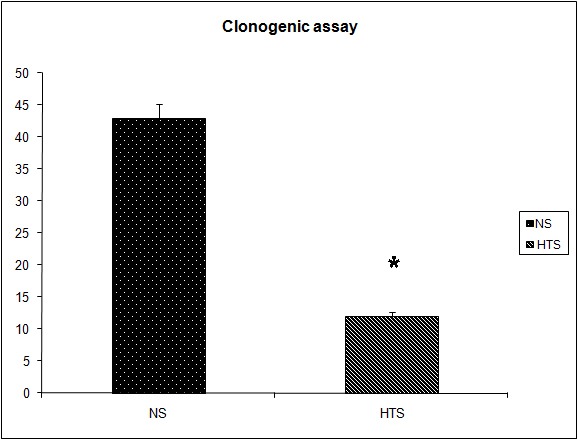
Effect of hypertonicity on clonogenic assay. Animals were injected with 5 x 10^4^ 4T1 tumor cells. Two minutes later mice received an intravenous bolus of 2 mL/kg normal saline (0.9% sodium chloride; NS) or 2 mL/kg hypertonic saline (7.5% sodium chloride; HTS) via lateral tail vein. Animals were sacrificed after 14 days and clonogenic assay performed. Data are expressed as mean and are representative of n = 20 animals. ^*^P < 0.05 versus NS control.

It was observed that the induction of a transient systemic hypertonicity (peek of 397 mOsm systemically at 30 minutes) resulted in a significantly reduced metastatic burden as quantified by the number of lung nodules (8 versus 4, P = 0.004, Fig. 4) and the lung/body weight ratio which approached significance (12.95 versus 11.7, P = 0.06, Fig. 5). This effect was further evident upon the culturing of tumor cells from the lungs retrieved post mortem, and cultured as colonies in a clonogenic assay. The number of clones cultured in the hypertonic group was significantly less than those in the group treated with normal saline (43 versus 12, P = 0.04, Fig. 6). The induction of a transient systemic hypertonicity reduced the lung/body weight ratio following stimulation with LPS (15 versus 13, P = 0.08), however this was not statistically significant (Fig. 5). There were no unexpected mortalities during this experiment.

## Discussion

The invasion process involves a number of steps necessary for effective tumor metastases including multiple interfaces between cells, extra cellular matrix, endothelium and the basement membrane [31]. LPS increases tumor cell metastases and metastatic growth [12, 15, 16]. This study supports the finding that LPS directly influences tumor cell invasion in vitro, with a 27% increase in tumor cell invasion. Tumor cells stimulated in the presence of hypertonicity did not show the increase in invasiveness seen in the control group. Instead the cells in a hypertonic environment retained the invasive capacity of unstimulated cells. It is clear from this, that while a hypertonic environment does not have the effect of reducing basic tumor cell invasion, it does negate the mechanisms activated through LPS stimulation. Stimulation of tumor cells with LPS results in the activation of NFκB [32], a signalling pathway strongly associated with tumor progression [33]. A hypertonic environment blocks this NFκB activation [20], suggesting a possible mechanism for the reduction in invasion demonstrated in this study.

The effect of MMPs on the ability of tumor cells to invade is clear [5-9], however the relative importance of tumor cell or host expressed MMPs, remains quite controversial. In this series of experiments cells cultured in a hypertonic environment exhibited just 1% of the proMMP-9 expression seen in control cells. The stimulation of tumor cells with LPS resulted in a profound increase in proMMP-9 expression, an effect completely negated by a hypertonic environment. Thus, a hypertonic environment can successfully attenuate the effects of LPS on proMMP-9 expression in vitro.

Previous authors have clearly demonstrated a link between cell volume and proliferation [34]. Additionally, a change in the cytoskeleton of cells induced by external osmotic forces may help explain the shedding of surface selectins and integrins. We have demonstrated a clear alternation in the nuclear/cytoplasmic ratio following exposure to a hypertonic environment, resulting in a smaller total cell volume. This phenomenon may explain the reduction in proliferation and integrin expression. The shedding of L-selectin plays an important role in limiting inflammation and microvascular leakage. Given the growing body of evidence to suggest that inflammation, such as that seen after surgery, drives tumor growth [13, 14], it is interesting to postulate that this shedding of L-selectin may play a role in attenuating the pro metastatic affects of the inflammatory process [35].

The use of HTS to produce a hyperosmotic state has reduces mortality and morbidity associated with sepsis [20] and pancreatitis [22]. It has been used successfully in the resuscitation of patients following trauma [23, 24]. In this study we have demonstrated that a hyper-osmotic state can reduce the metastatic load following IV dissemination of tumor cells. Through our in-vitro studies we can postulate that this effect is mediated by the cytoskeletal changes induced in tumor cells following exposure to a hypertonic environment. These changes result in a profound disruption of the cells metastatic ability. The loss of expression of the β1-integrin unit severely limits its adherence capacity. Additionally, studies have shown the importance of the MMP family of proteases to the metastatic process, particularly MMP-9. We have demonstrated that a hypertonic environment reduces the expression of proMMP-9 from 4T1 tumor cells, further explaining the in-vivo results described here. With such a profound effect on the expression of β1-integrin (as previously shown) and MMP-9 it is not surprising that tumor cell metaststatic actvity is reduced in a hypertonic environment. This temporary osmotic ‘stunning’ of tumor cells may render them susceptible to the host defence, resulting in a diminished metastatic burden.

As discussed, the administration of LPS has been shown to promote both tumor growth and metastases. This study has demonstrated that these affects can be attenuated through the creation of a transient systemic hypertonicity. In the model used in this study tumor cells were administered followed by LPS 1 week later. The intervention group were administered HTS within 5 minutes of LPS. This resulted in a significantly reduced lung/body weight ratio and accordingly tumor volume, when the animals were culled 1 week later. As expected, the number of lung nodules was not affected, showing that the number of tumor cells seeded were similar between both groups.

A limitation of this study is its use of only in vitro and animal models. Similarly it examined only a small portion of the myriad of factors that influence tumour cell metastases. However it does raise interesting possibilities for the manipulation of tonicity as a potential medical tool. In the clinical setting these effects may potentially protect against the initial pro metastatic effects of excisional surgery. Furthermore, the protective properties of hypertonicity following exposure to endotoxin, may defend against its tumor promoting affects following contamination of the surgical field, as seen in perforated or obstructing bowel tumors.
